# Molecular and neural circuit mechanisms of parvalbumin (PV) neurons in depression: Insights and advances

**DOI:** 10.1016/j.isci.2026.116454

**Published:** 2026-06-17

**Authors:** Zhi-xiao Li, Tian-en Si, Qi-rui Wu, Jia-heng Zhao, Wen-zhe Hua, Ying-ying Zhang, Sitian Yang, Yuan-yuan Mao, Wei-dong Zang, Jing Cao

**Affiliations:** 1 Department of Anatomy, Basic Medical College, Zhengzhou University, Zhengzhou 450001, China; 2 The First Affiliated Hospital of Zhengzhou University, Zhengzhou, Henan, China

**Keywords:** Health sciences, Medicine, Medical specialty, Psychiatry, Natural sciences, Biological sciences, Neuroscience

## Abstract

Interest in parvalbumin (PV) neurons has surged in recent years, driven by new insights into their role in the pathogenesis of depression. As a key class of γ-aminobutyric acid (GABA)-ergic interneurons, PV neurons have emerged as critical regulators of excitatory-inhibitory (E/I) balance, a core mechanism disrupted in depression. Growing evidence links PV neuron dysfunction to mood disturbances and cognitive impairments in depression. Recent studies highlight molecular alterations in PV neurons, including gene regulation, epigenetic modifications, and neurotransmitter/receptor imbalances, as well as circuit dysfunctions in the medial prefrontal cortex, hippocampus, and amygdala in depression. Interventions targeting PV neurons, such as pharmacological agents, neurotrophic factor modulation, and neuromodulation, show promise in restoring function. This review summarizes current advances in the molecular and circuit mechanisms of PV neuron dysfunction in depression, with the aim of providing a useful reference for future related studies.

## Introduction

Depression is a mental disorder characterized by persistent low mood and anhedonia, accompanied by cognitive, behavioral, or autonomic disturbances.^1^^,^^2^ An estimated 280 million people worldwide (approximately 3.8% of the global population) suffer from depression, with women suffering about 50% more than men. Globally, over 10% of pregnant women and postpartum women suffer from depression.^3^ Unlike normal mood swings, depression is characterized by a prolonged duration, high recurrence rates, and intermittent relief. It not only impairs physical and mental health, causing fatigue, sadness, and apathy, and leading to weight loss, sleep disorders, and even suicide,^4^ but also significantly impairs patients’ social functioning, imposing a substantial economic and medical burden on individuals, families, and society.^5^ The etiology of depression is highly heterogeneous, resulting from a combination of social, psychological, and biological factors. Several susceptibility factors contribute to its development, including genetic susceptibility, environmental stressors, early-life trauma, and neurobiological imbalances.^6^ Treatment for depression is typically selected according to symptom severity, clinical need, and patient preference, with psychotherapy, antidepressant medication, or their combination recommended as standard options.^7^ Despite these established treatment strategies, clinical outcomes remain suboptimal, and significant challenges persist in the management of depression.^8^

Importantly, depression is increasingly understood as a disorder involving abnormalities across multiple levels, from molecular and cellular alterations to circuit dysfunction and large-scale network imbalance. In this context, a cross-level framework linking cortical GABAergic dysfunction to network-level abnormalities and depressive psychopathology has been proposed,^9^ while more recent work further emphasized the importance of excitation-inhibition (E/I) imbalance in depression.^10^ γ-aminobutyric acid (GABA), the principal inhibitory neurotransmitter in the central nervous system, plays an essential role in maintaining neural network stability and regulating the E/I balance. Dysregulation of GABAergic signaling frequently leads to E/I imbalance, which has been implicated in the pathophysiology of numerous psychiatric and neurological disorders, including depression.^11^ Clinical studies have demonstrated significantly reduced GABA levels and diminished GABAergic synaptic transmission in cerebrospinal fluid, plasma, occipital cortex, prefrontal cortex (PFC), and anterior cingulate cortex (ACC), as measured by proton magnetic resonance spectroscopy in patients with major depressive disorder (MDD).^12^^,^^13^

**Figure 1:**
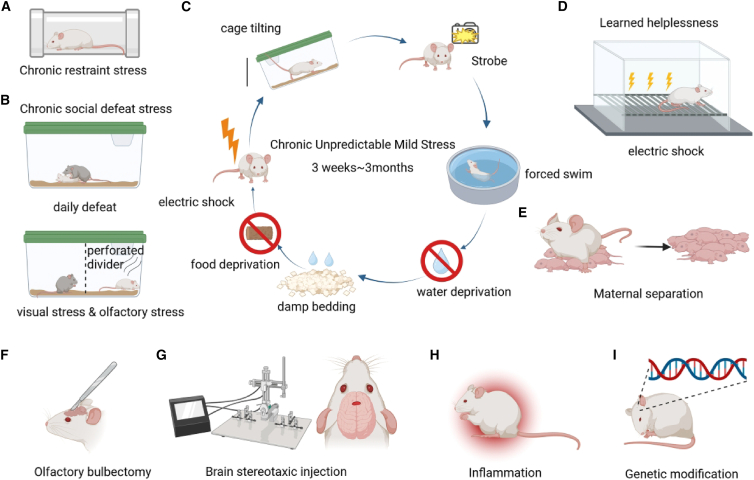
Animal models for the study of depressive disorder and their relevance to PV neuron dysfunction (A) Chronic restraint stress (CRS) – repeated physical restraint induces sustained stress-related behavioral and neuroendocrine changes via HPA axis activation and has been associated with reduced PV expression in the prefrontal cortex. (B) Chronic social defeat stress (CSDS) – repeated exposure to social stress induces susceptible and resilient phenotypes and has been associated with impaired claustrum-to-prelimbic cortex input to PV neurons and increased activity of ventral pallidal PV neurons, thereby contributing to social avoidance and despair-like behaviors. (C) Chronic unpredictable mild stress (CUMS): Repeated exposure to variable mild stressors induces anhedonia-like and depression-like behaviors and has been associated with increased PV expression and PV-positive cell number in the mPFC, suggesting altered PV-mediated inhibition and disrupted prefrontal E/I balance. (D) Learned helplessness (LH) – exposure to uncontrollable stress impairs active coping behavior and has been associated with reduced PV neuron excitability and weakened GABAergic transmission. (E) Maternal separation/early-life stress: Disruption of early postnatal maternal care induces long-lasting neurodevelopmental changes and has been linked to impaired or delayed PV neuron maturation and reduced PNN integrity, contributing to weakened inhibitory circuit stability in emotion-related brain regions. (F) Olfactory bulbectomy (OBX) – surgical removal of the olfactory bulbs disrupts olfactory-limbic communication and reduces limbic gamma oscillations, thereby contributing to depression-related behavioral abnormalities. (G) Post-stroke depression – ischemic brain injury induces neuroinflammation, impaired synaptic plasticity, and corticolimbic network dysfunction, resulting in secondary depression-related phenotypes. (H) Inflammatory models (e.g., LPS) – systemic immune activation induces neuroinflammation-related depression-like behaviors and activates NF-κB signaling, causing PV neuron dysfunction and gamma oscillation deficits. (I) Genetic modeling – targeted manipulation of depression-related genes, such as glucocorticoid receptor or CREB, enables causal dissection of how HPA-axis regulation and neurotrophic signaling contribute to depression-related phenotypes.

GABAergic interneurons are classified into three major non-overlapping classes based on the expression of specific molecular markers, electrophysiological properties, and morphological characteristics^14^ (1) Parvalbumin (PV)-expressing neurons account for approximately 40% of cortical interneurons and include basket cells and chandelier cells. These neurons exhibit fast-spiking (FS) firing patterns and primarily target the perisomatic region of pyramidal neurons. (2) Somatostatin (SST)-expressing neurons represent about 30% of interneurons, primarily include Martinotti cells, and target the distal dendrites of pyramidal neurons. (3) 5-HT3a receptor-expressing neurons constitute the remaining 30% and include vasoactive intestinal peptide (VIP)-expressing interneurons as well as other subtypes that do not express PV or SST. Among these, PV neurons represent a critical class of inhibitory neurons in both humans and rodents, including both local circuit interneurons in cortical regions and projection neurons in certain subcortical structures such as the thalamic reticular nucleus (TRN), where they provide inhibitory projections to thalamic relay nuclei.^15^ PV neurons are characterized by their FS phenotype, low input resistance, and high-amplitude rapid after-hyperpolarization, making them essential for regulating inhibitory neural networks.^15^ These neurons mediate both feedforward and feedback inhibition of pyramidal neurons.^16^ Relevant evidence suggests that reduced numbers or dysfunction of PV neurons are associated with a range of neuropsychiatric and neurological disorders, including depression.^17^ Specifically, dysfunction of PV neurons is linked to disturbances in gamma (γ) oscillatory rhythms and excitation-inhibition (E/I) imbalance.^18^^,^^19^^,^^20^ These abnormalities can impair inhibitory signaling in the mPFC, amygdala, and other regions implicated in depression, contributing to the mood and cognitive symptoms characteristic of the disorder.^21^ However, many patients show inadequate responses to current therapies, particularly in cases of treatment-resistant depression.^22^ Therefore, a deeper understanding of the molecular mechanisms and neural circuits involving PV neurons in depression is crucial for the development of more effective treatments. In addition, we summarize major animal models of depression-related phenotypes in Figure 1, which provides conceptual background for the molecular and circuit-level mechanisms discussed later in discussion.

## Molecular mechanisms

Different molecular mechanisms influence PV neuron function. These mechanisms involve not only alterations in gene expression, epigenetic regulation, and signaling pathways, but also imbalances in neurotransmitter systems. A deeper understanding of these molecular processes may help to elucidate the role of PV neurons in depression and provide novel insights for future therapeutic strategies.

### Gene expression and epigenetic regulation

**Figure 2:**
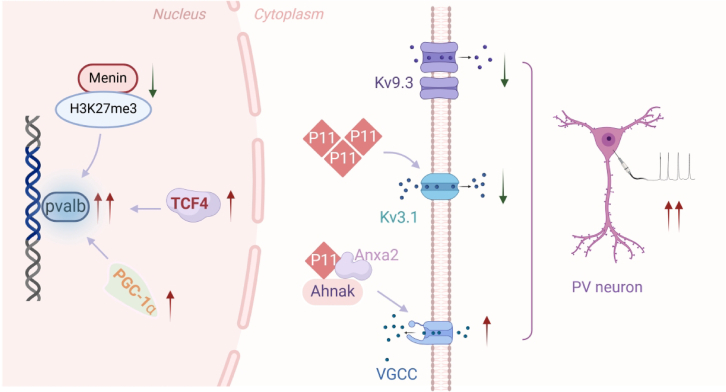
Epigenetic Regulation and Molecular Mechanisms in PV Neurons This schematic illustrates the complex molecular and epigenetic mechanisms regulating parvalbumin (Pvalb, PV) neuron function. Menin deficiency in PV neurons promotes H3K27me3 histone modifications, leading to increased transcription of the Pvalb gene and subsequent elevation in PV expression. Similarly, Transcription factors TCF4 and PGC-1α also upregulate Pvalb expression, enhancing PV neuron function. In the cytoplasm, the regulatory protein P11 (S100A10) plays a pivotal role in modulating ion channel activity. P11 interacts with Kv3.1 and Kv9.3 potassium channels, which govern neuronal firing properties: Kv3.1 facilitates rapid repolarization, enabling high-frequency firing, while Kv9.3 aids in maintaining hyperpolarization. Additionally, P11, in association with Ahnak and Annexin A2 (Anxa2), regulates voltage-gated calcium channels (VGCCs), controlling calcium influx essential for neurotransmitter release and excitability. These epigenetic and molecular pathways collectively fine-tune PV neuron activity and their role in neural network synchronization.

Current evidence suggests that PV-related alterations in depression are complex. Human postmortem evidence for PV alterations in MDD is limited and heterogeneous across brain regions. In the dorsolateral PFC, some studies have reported no clear change in PV-positive cell density, whereas others found a reduction in PV immunostaining in layer VI. In the orbitofrontal cortex, reduced PV cell density has been reported. In addition, reduced PV-related gene expression has been described in the subgenual ACC.^23^ Findings from animal models are likewise heterogeneous. PV-related abnormalities in depression may involve different brain regions, experimental models, and disease stages. Importantly, reported changes in PV-positive cell density may reflect actual loss of PV interneurons, reduced PV protein expression, or a combination of both. Therefore, understanding how PV transcription and expression are regulated may help explain the heterogeneous PV-related findings observed in depression. In this section, we summarize current evidence on the gene expression and epigenetic regulation of PV neurons in depression, with the specific mechanisms illustrated in Figure 2.

#### Epigenetic mechanisms and PV neurons in depression

Epigenetic mechanisms, including deoxyribonucleic acid (DNA) methylation, histone modifications, and non-coding RNAs (ncRNAs), play a crucial role in regulating depression-like behaviors. Among them, histone modifications are strongly linked to the expression of PV in depression. The Multiple Endocrine Neoplasia Type 1 (MEN1) gene in humans (*Men1* in mice) encodes the scaffold protein Menin.^24^^,^^25^ As a nuclear scaffolding protein, Menin can induce chromatin condensation through histone modifications, leading to the repression of gene transcription.^26^ Importantly, *Men1* deficiency in PV neurons is directly linked to depressive phenotypes. *Men1*-deficient mice in PV neurons (PcKO) exhibit increased cortical PV expression and depressive-like behavior. This mechanism is due to Menin defects in PV neurons, which promote increased PV transcription through Histone H3 Lysine 27 Trimethylation (H3K27me3) modification. The depressive-like behavior of PcKO mice can be ameliorated by restoring Menin, knocking down PV expression, or inhibiting PV neuronal activity.^27^ DNA methylation also plays a significant role in depression. In patients with MDD, abnormal methylation of the *Pvalb* promoter has been reported and may be associated with symptom severity.^28^ In preclinical studies, early life stress (Figure 1) may increase the risk of depression by affecting 5-methylcytosine (5-mC) DNA methylation in PV neurons in the PFC, with potential gender-specific effects.^29^

#### Transcription factors and PV neurons in depression

Transcription factor 4 (TCF4) is a basic-helix-loop-helix transcription factor implicated in various psychiatric disorders, including MDD.^30^ Single-cell sequencing has demonstrated that TCF4 is highly enriched in PV neurons. And mutations in the *Tcf4* gene in mouse models lead to a reduction of PV neurons in the cortex.^31^ The loss of PV neurons disrupts the balance between excitatory and inhibitory inputs to pyramidal neurons in the cortex, which impairs cortical network function. These data suggest that TCF4 is not only an important transcription factor regulating the development of PV neurons but also that its aberrant function may significantly affect the pathophysiological processes of depression. The transcriptional coactivator peroxisome proliferator-activated receptor γ coactivator 1α (PGC-1α) facilitates the interaction of transcription factors with their activator proteins, thereby forming functional DNA-binding complexes^32^ Thus, its downstream targets depend on the transcription factors it co-expresses. In PGC-1α knockout mice, PV neuron expression is significantly reduced throughout the brain, while overexpression of PGC-1α in cells is sufficient to strongly induce PV expression.^33^ In the hippocampus, PGC-1α deficiency leads to a reduction in the number and activity of PV neurons in the CA3 region of the hippocampus. However, physical exercise can modulate PV neurons in the hippocampus through PGC-1α, thereby reversing depressive-like behavior.^34^ Taken together, these findings suggest that PGC-1α plays a critical role in regulating gene expression and function in PV neurons and may serve as a promising therapeutic target for depression.^35^

#### Ion channel expression and PV neuron in depression

The unique FS phenotype of PV neurons depends on the expression of multiple subtypes of voltage-gated potassium channels (Kv).^36^ PV neurons in the PFC selectively express *Kcns3*, which encodes the Kv9.3 potassium channel. This channel is crucial for maintaining neuronal excitability and synaptic transmission.^37^ Deletion of the *Kcns3* gene impairs the firing of PV neurons, destabilizing neural network function, which may contribute to psychiatric disorders.^38^ Additionally, the expression of potassium channels in PV neurons is regulated by other factors. The protein p11 (also known as S100A10), a member of the S100 calcium-binding protein family.^39^ Conditional knockdown of p11 in DG PV neurons reduces Kv3.1 expression and attenuates the high-frequency firing capacity of these cells, changes that are associated with increased vulnerability to depression-related behavior under stress. Conversely, overexpression of p11 in DG PV neurons, as well as upregulation or acute pharmacological activation of Kv3.1 channels, has been reported to promote behavioral resilience.^40^

In addition, L-type voltage-gated calcium channels (VGCCs) have been implicated in various psychiatric disorders, including depression.^41^ L-type VGCCs act as downstream effectors of the Ahnak/p11/Anxa2 complex.^42^ Ahnak, a large (700 kDa) structural scaffolding protein, plays a critical role in the formation of the α1 subunit of L-type VGCCs via its N-terminal region, and facilitates the assembly of the β-subunit of VGCCs with the p11/Anxa2 complex through its C-terminal region.^43^^,^^44^ Forebrain glutamatergic neuron-selective Ahnak KO mice exhibit depressive-like phenotypes, including deficits in pleasure responses, similar to the behavioral profiles of p11 knockout mice. In contrast, a reduction of L-type calcium currents in PV neurons and the emergence of antidepressant-like behaviors were observed in PV neuron-selective Ahnak KO mice.^42^^,^^45^ This reveals a novel molecular link between L-type VGCCs as effectors of the Ahnak/p11/Anxa2 complex involved in the control of depressive behavior.

### Neurotrophic factors and signaling pathways

**Figure 3:**
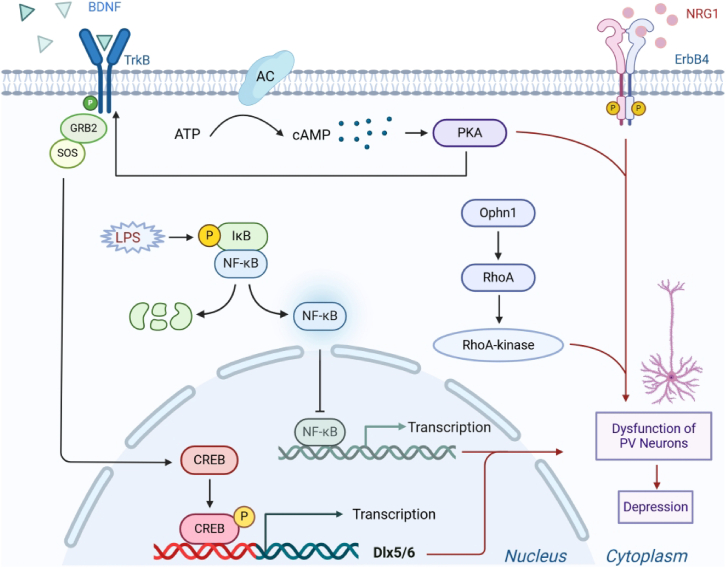
Neurotrophic factors and signaling pathways involved in the regulation of PV neurons This figure summarizes the intricate molecular mechanisms regulating the function and plasticity of parvalbumin (PV) interneurons, which are implicated in mood disorders such as depression. The BDNF-TrkB signaling pathway regulates PV neuron activity and synaptic plasticity by modulating CREB phosphorylation and downstream transcription of Dlx5/6. The NRG1-ErbB4 pathway plays a critical role in PV neuron development, synaptic integration, and circuit homeostasis. Additionally, the AC-cAMP-PKA axis modulates synaptic strength and neurotransmitter release, further supporting PV neuron activity. However, chronic inflammation activates the NF-κB pathway, disrupting gene transcription and synaptic transmission, ultimately leading to PV neuron dysfunction. The convergence of these mechanisms underscores the pivotal role of PV neurons in maintaining neural circuit stability and highlights their dysfunction as a potential contributor to depressive disorders.

Notably, the BDNF/TrkB, NRG1-ERBB4, and cAMP-PKA-CREB signaling pathways have been implicated in the pathophysiology of depression. Research suggests that chronic stress triggers the dysregulation of these pathways, leading to E/I imbalance in the brain by affecting the function of PV neurons. This imbalance, in turn, affects mood and cognitive functioning, potentially serving as key factors in the development of depression. The mechanism of signaling pathway effects on PV neurons in depression is shown in Figure 3.

#### The BDNF-TrkB signaling pathway

Signaling through brain-derived neurotrophic factor (BDNF) and its receptor, tropomyosin receptor kinase B (TrkB), is essential for synaptic plasticity, stress adaptation, and cognitive function.^46^^,^^47^ Chronic stress has been shown to reduce BDNF mRNA levels in several depression-relevant brain regions, including the PFC, hippocampus, and thalamus. Reduced BDNF/TrkB signaling may further downregulate GABAergic markers and impair inhibitory circuit function, thereby contributing to depression-like behaviors.^48^^,^^49^ Furthermore, decreased BDNF levels can impair the function of PV neurons within hippocampal microcircuits and disrupt gamma rhythm network activity, which can contribute to memory deficits.^50^

BDNF/TrkB signaling regulates PV neurons through multiple cellular mechanisms. Acute BDNF application has been reported to inhibit excitatory postsynaptic currents (EPSCs) in most PV neurons, an effect blocked by anti-TrkB IgG, indicating that this acute action is mediated through TrkB.^51^ In PV neuron-specific TrkB knockout mice, loss of TrkB disrupts PV neuron function and leads to memory impairments, highlighting the importance of TrkB signaling in maintaining PV-dependent cognitive processes.^52^^,^^53^ In addition, TrkB activation in PV neurons promotes the phosphorylation of Kv3.1 channels and reduces their intrinsic excitability, suggesting that BDNF/TrkB signaling can tune PV neuron firing properties and coordinate cortical network activity.^54^

Beyond neurotrophic signaling, transcriptional regulators involved in the development and maintenance of PV neurons may also contribute to depression-related PV-neuron dysfunction. Distal-less Homeobox 5 and 6 (Dlx5/6) are key transcription factors involved in the development, differentiation, and maintenance of PV neurons.^55^ Elevated Dlx5/6 expression has been associated with alterations in cortical PV neurons in animal models showing depression-like behaviors.^56^ Notably, fluoxetine has been reported to modulate PV-related inhibitory plasticity through a TrkB-dependent pathway involving the inhibition of Dlx5/6 in cortical GABAergic neurons, while repeated treatment also induces long-lasting neurotrophic changes in the medial PFC.^57^^,^^58^

Together, BDNF/TrkB signaling regulates PV neuron excitability, synaptic function, and transcriptional programs. Its dysregulation may contribute to PV-neuron dysfunction in depression, while antidepressant-induced TrkB activation may help restore inhibitory circuit function.

#### NRG1-ErbB4 signaling pathway

Neuregulin-1 (NRG1) is an important neurotrophic factor that regulates the development, maturation, and synaptic function of PV neurons by binding to its receptor, epidermal growth factor receptor-4 (ErbB4) .^59^^,^^60^ It has been shown that the absence of ErbB4 signaling leads to reduced excitatory synaptic inputs and decreased activity in PV neurons, thereby disrupting the E/I balance in the brain.^61^ Abnormalities in ErbB4 splicing have been observed in both schizophrenia, with evidence linking such splicing alterations to PV neuron dysfunction.^62^^,^^63^ Aberrant ErbB4 splicing results in altered neural circuit function, particularly impairments in gamma oscillations and neural network synchronization associated with PV neurons.^64^^,^^65^ These disruptions can negatively impact cognitive and emotional processing, contributing to the development of depression and other psychiatric symptoms.^66^

Certain studies suggest that fetal stress (e.g., prenatal exposure to the glucocorticoid drug dexamethasone) may lead to the abnormal development of hippocampal PV neurons in adulthood by disrupting the NRG 1-ErbB 4 signaling pathway, which is linked to the emergence of anxiety- and depression-like behaviors in adulthood.^67^ This finding suggests that disturbances in NRG1-ErbB4 signaling during early developmental stages may have lasting effects on PV interneuron maturation and may contribute to later emotional and cognitive dysfunction. Given that PV interneurons undergo a relatively prolonged developmental process, they may be particularly vulnerable to prenatal and early postnatal stress. Apart from prenatal stress, other early-life stress paradigms also support this developmental framework. Maternal separation (Figure 1) has been reported to induce reduced PV expression and decreased PV-positive interneuron density in the PFC.^68^^,^^69^ Moreover, juvenile stress may alter perineuronal net formation around PV neurons,^70^ suggesting that early adversity can affect not only PV neuron activity but also the molecular and extracellular mechanisms required for their maturation.

In addition to its role in stress-related mood regulation, the NRG1-ErbB4 pathway has also been implicated in the rapid antidepressant effects of ketamine.^71^ Recent studies have shown that ketamine downregulates NRG1 expression in PV neurons in the mPFC of mice, corresponding to reduced synaptic inhibition onto excitatory neurons in the mPFC. This effect is blocked by PV-targeted ErbB4 receptor knockout, suggesting that ketamine may mediate cortical disinhibition through PV-specific NRG1 signaling.^72^ Collectively, these findings highlight the importance of NRG1-ErbB4 signaling not only in depression-related pathology but also in the developmental regulation and functional plasticity of PV interneurons.

#### cAMP-PKA signaling pathway

The adenylyl cyclase-cyclic adenosine monophosphate-protein kinase A (AC-cAMP-PKA) signaling pathway is critical for regulating the function of PV neurons, particularly in neuropsychiatric disorders such as depression. Reduced levels of cAMP and PKA activity were observed in brain regions associated with depression, such as the PFC and hippocampus. This reduction impaired the strength and inhibitory control of synaptic inputs exerted by PV neurons on excitatory circuits.^73^^,^^74^ For example, histamine 3 receptor (H3R) is differentially expressed at inhibitory synapses from cortical PV neurons to the nucleus accumbens (NAc). Presynaptic H3R activation reduces feedforward inhibition in PV neurons by inhibiting AC-cAMP-PKA signaling, thereby inducing depression.^75^^,^^76^ In addition, PV neurons are key regulators of gamma oscillations and network synchronization. Reduced activity of the cAMP-PKA pathway disrupts the plasticity of PV neurons, leading to the dysregulation of cortical rhythms and the manifestation of depression ,such as hopelessness.^77^^,^^78^ Whereas the inhibition of AC and PKA blocked the effects of propofol.^79^ In addition, the activation of the cAMP-PKA pathway in the amygdala inhibits GABA release from PV interneurons, leading to hyperexcitability and dysfunction of downstream neurons.^80^ In summary, activation and inhibition of the cAMP-PKA signaling pathway have similar effects on the excitability of PV neurons in different brain regions and experimental conditions. However, this does not mean that the two are contradictory, but rather reflects the complexity and brain region specificity of the cAMP-PKA signaling pathway in neural circuits.

Given the role of the cAMP-PKA pathway in the pathophysiology of depression, several targeted antidepressant strategies have been proposed.^81^ For instance, inhibition of phosphodiesterase-4 (PDE4), which degrades cAMP, has been shown to alleviate depressive-like behaviors by enhancing cAMP-PKA signaling.^82^^,^^83^ Ketamine restores the inhibitory control to excitatory neurons by enhancing cAMP-PKA pathway activity in PV neurons.^84^^,^^85^ Moreover, activation of the cAMP-PKA pathway can upregulate BDNF signaling via its receptor TrkB, which is essential for PV neuronal function.^86^^,^^87^ This suggests that modulating this pathway may alleviate depression-like behaviors by restoring PV neuron function and overall network stability.

#### NF-κB signaling pathway

NF-κB (NF-κB) is a crucial transcription factor involved in regulating the inflammatory response, cell survival, neuronal stress responses, and neurotrophic factor expression.^88^ Chronic inflammation can induce depression-like behaviors, with the NF-κB signaling pathway playing a key role in this process.^89^ The NF-κB pathway contributes to the pathophysiology of depression by promoting inflammation and disrupting normal signaling in neurons, particularly by impairing PV neuron survival and synaptic plasticity.^90^ Esketamine has been reported to reverse the reduction in PV neuron density and restore synaptogenesis in the inflammation-disturbed mPFC, possibly through BDNF/TrkB signaling and attenuation of the NF-κB pathway, thereby improving anesthesia- and surgery-induced depression-like symptoms.^91^

#### The Rho-GTPase signaling pathway

Oligophrenin-1 (OPHN1) is a Rho-GTPase-activating protein (Rho-GAP) that plays a crucial role in regulating synaptic function and neuronal network formation.^92^^,^^93^ Mutations or dysfunction of Oligophrenin-1 have been linked to X-linked intellectual disability, as well as other cognitive impairments and mental disorders.^94^^,^^95^^,^^96^ Correlative studies have shown OPHN1 deficits in PV neurons of the mPFC, resulting in reduced inhibitory output and overactivity of the prelimbic cortex (PrL), which induces depressive-like behavior.^97^ The Rho-GTPase signaling pathway is critical for PV neuron function and synaptic plasticity, influencing the excitatory-inhibitory (E/I) balance in the brain, which is involved in modulating depressive behavior.

### Neurotransmitter and receptor imbalances

**Figure 4:**
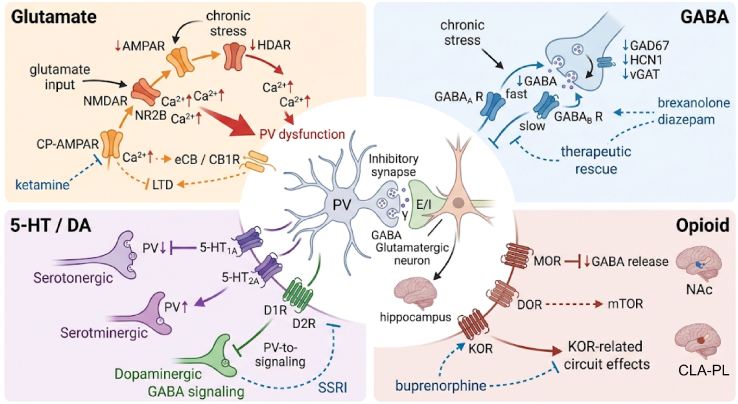
Various receptors on PV neurons are associated with depression AMPA receptors (AMPARs) and NMDA receptors (NMDARs), activated by glutamate (Glu, dark blue), facilitate sodium (Na+) and calcium (Ca2+) influx, respectively, contributing to excitatory postsynaptic potentials. AMPA receptors (AMPAR) and NMDA receptors (NMDAR) mediate excitatory synaptic transmission driven by glutamate (Glu, dark blue). AMPAR facilitates rapid excitatory signaling via sodium (Na+) influx, while NMDAR mediates calcium (Ca2+) signaling, critical for long-term synaptic plasticity. Dysregulation of AMPA and NMDA signaling in depression may impair PV neuron function, disrupting the excitation-inhibition (E/I) balance. GABA type A receptors (GABAAR) and type B receptors (GABABR) are activated by GABA (yellow). GABAAR mediates chloride (Cl-) influx for fast inhibition, whereas GABABR modulates downstream pathways, including reducing cyclic AMP (cAMP) levels and presynaptic calcium influx. μm-opioid receptors (MOR) and κ-opioid receptors (KOR) regulate synaptic activity by reducing cAMP levels and activating GIRK channels, modulating GABA and glutamate release. Dopamine receptors (DR) and serotonin receptors (5-HTR) mediate signaling of dopamine (DA, pink) and serotonin (5-HT, gray), respectively, via G-protein-coupled receptor (GPCR) mechanisms. These receptor systems highlight the complex integration of excitatory, inhibitory, and neuromodulatory inputs that regulate postsynaptic neuronal activity and contribute to circuit-level modulation.

In depression, PV neurons are influenced by various neurotransmitters and their receptors, which are crucial for regulating the excitation-inhibition balance in the brain. Neurotransmitter imbalances (especially in the GABA, 5-hydroxytryptamine, and dopamine (DA) systems) and synaptic plasticity impairments in depressed patients directly disrupt the function of PV neurons (Figure 4).

#### Glutamate and its receptors

Glutamate is the main excitatory neurotransmitter in the central nervous system, responsible for regulating excitatory transmission between neurons. N-methyl-D-aspartate receptors (NMDARs) and α-amino-3-hydroxy-5-methyl-4-isoxazolepropionic acid receptors (AMPARs) are the two main subtypes of glutamate receptors that regulate neuronal excitability and synaptic plasticity by respectively modulating the influx of calcium and sodium ions.^98^ PV neurons are regulated by glutamate through NMDARs and AMPARs on their surface.^99^^,^^100^ Activation of glutamate receptors enhances PV neuron responsiveness to their environment, thereby promoting the synchronization of brain networks.^20^ Repetitive stress significantly reduces the expression of AMPARs in the PFC, disrupting the balance between glutamate and GABA signaling, inhibiting PV neuron excitability, and leading to E/I imbalance of local networks.^101^ AMPARs-specific knockout in the PFC of mice, on the other hand, induces depressive-like behavior.^102^ Furthermore, evidence suggests that the activation of AMPARs in the PFC is essential for the antidepressant effects of deep brain stimulation (DBS).^103^ Notably, PV neurons selectively express calcium-permeable AMPARs (CP-AMPARs) that lack the GluA2 subunits.^104^^,^^105^ It has been shown that the activation of CP-AMPARs on PV neurons in the hippocampus and NAc triggers presynaptic cannabinoid receptor 1 (CB1R)-dependent endogenous cannabinoid (eCB) signaling by mediating calcium influx, leading to long-term depression (LTD), which reduces PV neuron responsiveness to subsequent excitatory stimuli.^106^^,^^107^ In summary, alterations in AMPARs function in depression may impact their activity by influencing excitatory inputs to PV neurons, synaptic plasticity, and the homeostasis of neural network dynamics.

Under physiological conditions, NMDAR-mediated signaling in PV neurons helps maintain inhibitory control and supports normal circuit function. However, under chronic stress or pathological conditions, excessive glutamatergic activity may lead to abnormal calcium influx through NMDARs, thereby impairing PV neuron function and disrupting neural network balance.^19^^,^^108^ In animal studies, chronic stress has been reported to induce sex-specific increases in NR2B-containing NMDAR expression in PV neurons in the mPFC, which have been associated with altered excitability and synaptic plasticity, accompanied by anxiety- and depression-like behaviors.^109^ These findings suggest that stress-induced changes in NMDAR signaling may shift PV neurons from a physiological role in circuit synchronization toward maladaptive regulation of prefrontal microcircuits. However, this relationship is not universal. Notably, mice lacking NMDARs specifically in PV neurons display normal depression-related behaviors and retain responsiveness to the antidepressant-like effects of NMDAR antagonists.^110^ Antagonists of NMDARs, such as ketamine, have been shown to produce rapid antidepressant effects. Systemic ketamine administration not only alters PV expression and reverses depressive-like behavior,^111^ but also protects PV neurons and restores normal excitation-inhibition balance in the brain by blocking NMDARs-mediated excitotoxicity.^100^^,^^112^ Together, these findings support the view that NMDAR-dependent signaling in PV neurons is an important component of stress-related circuit dysfunction, but its involvement in depression is nuanced and likely depends on broader network and cellular context.

#### GABA and its receptors

PV neurons release GABA and modulate target neuron excitability via GABA type A receptor (GABAAR) and GABA type B receptor (GABABR). In chronically stressed animals, PV-neuron-related GABAergic dysfunction has been associated with reduced GAD67 expression.^113^^,^^114^ Additionally, the functions of hyperpolarization-activated cyclic nucleotide-gated channel 1 (HCN1) and vesicular GABA transporter (vGAT) at the axon terminals of PV neurons are inhibited in depression and chronic stress, resulting in reduced GABA reserves and insufficient release, which in turn affect GABAergic neurotransmission.^115^^,^^116^ Reduced GABA levels decrease PV neuron inhibition of excitatory neurons, disrupting neural network homeostasis and impairing emotional regulation. To increase GABA availability, GABA uptake inhibitors, such as tiagabine, prolong the residence time of GABA in the synaptic cleft by inhibiting GABA transporter 1 (GAT-1).^117^ Moreover, combination neuromodulation techniques (e.g., transcranial magnetic stimulation (TMS)) and GABA-enhancing drugs may improve the E/I balance of neural networks, showing particular promise for patients with refractory depression.^118^

GABAARs are fast-responsive ionotropic receptors that regulate chloride ion (Cl-) flow, playing a key role in rapid feedback inhibition of surrounding excitatory inputs by PV neurons.^119^ This immediate inhibitory input is crucial for maintaining synchronization and stability in neural networks, particularly in the cortex and hippocampus.^120^ Early-life adverse events induce depressive behaviors, such as anhedonia, by downregulating GABAAR expression in the hippocampus.^121^^,^^122^ The loss of δ-subunit-containing GABAAR in PV neurons of the hippocampus during pregnancy alters γ-oscillation frequency, disrupting the activity of networks, which offers key insights into the mechanisms of postpartum depression.^123^ Brexanolone, a drug targeting the GABAAR, selectively modulates the δ-subunit and significantly alleviates mood dysregulation in depression. It has been approved for treating postpartum depression.^124^ GABABR is a G-protein-coupled receptor that mediates slower inhibitory synaptic responses, modulates LTD, which plays a crucial role in long-term E/I balance in neural networks.^125^^,^^126^ It was shown that the activation of presynaptic GABABR inhibits GABA release from PV neurons onto hippocampal pyramidal cells, with P/Q-type calcium channels on PV terminals involved in GABABR-mediated inhibition.^127^^,^^128^ Although GABAB receptor agonists, such as baclofen, have been shown to exert antidepressant effects, no definitive studies have yet identified how GABAB receptors on PV-positive neurons are altered in depression.^129^

In summary, depression and chronic stress reduce GABA levels in PV neurons, which is associated with decreased GAD67 expression and impaired transport mechanisms. Dysfunction of GABAAR and GABABR exacerbates network imbalances, contributing to depression-like behaviors.

#### 5-Hydroxytryptamine (5-HT) and dopamine (DA) systems

The abnormal activity of PV neurons is closely linked to the dysfunction of the 5-hydroxytryptamine (5-HT) and dopamine (DA) systems, which play a critical role in the neural mechanisms underlying stress responses and depression. Studies indicate that individuals with depression frequently exhibit the dysregulation of 5-HT transmission.^130^ In animal studies, 5-HT has been shown to modulate PV neuron activity through 5-HT1A receptors (5-HT1ARs) and 5-HT2A receptors (5-HT2ARs), which may differentially contribute to depression-like phenotypes.^131^ Upregulation of 5-HT1ARs in PV neurons reduces their excitability, attenuating their inhibitory effect on pyramidal neurons.^132^ Conversely, activation of 5-HT2ARs typically increases PV neuron excitability, enhancing the inhibition of pyramidal neurons. This decreases overall network excitability, a process that may help reduce memory deficits and stress-related behavioral abnormalities in animal models.^133^

The DA system also plays a crucial role in PV neurons in depression. Depressed individuals often exhibit reduced dopaminergic system function, particularly manifesting as symptoms such as anhedonia and lack of motivation.^134^ In addition, PV neurons in the PFC express high levels of DA D1 receptors (D1Rs) and DA D2 receptors (D2Rs) .^135^ DA can inhibit GABAergic signaling from PV neurons to pyramidal neurons through presynaptic D2Rs, affecting the E/I balance in PFC and exacerbating depression-like behaviors.^80^^,^^136^ Under chronic stress conditions, combined dysregulation of the 5-HT and DA systems may impair PV neuron function, disrupt affective regulation, and contribute to depression-like phenotypes in animal models.^137^ Whereas treatments targeting the 5-HT system, such as selective serotonin reuptake inhibitors (SSRIs), which increase the concentration of 5-HT in the synaptic gap, have demonstrated significant therapeutic effects on depression.^138^^,^^139^ Similarly, treatments that enhance dopaminergic transmission, such as norepinephrine-DA reuptake inhibitors and DA receptor agonist augmentation strategies, have been used or investigated for depression, particularly in patients with anhedonia, motivational deficits, or treatment-resistant symptoms.^140^^,^^141^ In summary, the 5-HT and DA systems play essential roles in the involvement of PV neurons in depression. By regulating the balance between these neurotransmitter systems, it is possible to influence PV neuron activity, thereby restoring the E/I balance of neural circuits, which improves mood regulation and cognitive function.

#### Cholinergic signaling

In addition to monoaminergic systems, cholinergic signaling should also be considered in the neuromodulatory control of PV neurons. Cholinergic signaling has been implicated in mood regulation and is associated with stress sensitivity, negative affective processing, and depression-like behaviors.^142^ Studies have shown that enhanced hippocampal acetylcholine signaling can increase anxiety- and depression-like behaviors and reduce resilience to social defeat stress, while activation of basal forebrain - ventral subiculum cholinergic projections can induce depression-like behaviors through muscarinic receptor-dependent mechanisms.^143^ In parallel, acetylcholine can regulate PV interneuron excitability and inhibitory output. In the hippocampus and PFC, M1 muscarinic receptor activation directly excites PV interneurons and can strengthen PV-related inhibitory control over pyramidal neurons.^144^^,^^145^ Thus, cholinergic regulation may represent an additional pathway through which PV interneurons influence E/I balance, gamma oscillations, and mood-related circuit function.

#### Opioid neurotransmitters and their receptors

The main receptor types in the opioid system include μ-opioid receptors (MORs), delta-opioid receptors (DORs), and κ-opioid receptors (KORs), each with distinct expression patterns and roles in PV neurons.^146^ Activation of MOR inhibits GABA release from PV neurons, reducing their inhibitory input to pyramidal neurons in the CA1 region of the hippocampus, which disrupts the E/I balance.^147^ Research has shown that morphine, acting via MOR on PV neurons in the PrL, decreases their inhibitory effects on pyramidal cells. Similarly, activation of DOR on PV neurons inhibits their inhibitory transmission in hippocampal regions.^148^ DOR agonists exert antidepressant effects by inhibiting GABA release from PV neurons through the mTOR signaling pathway, which enhances the excitation of the infralimbic cortex (IL)-PFC pyramidal neurons.^149^ Furthermore, morphine enhances the inhibitory effect of SST neurons on PV neurons by acting on DORs, leading to a disinhibitory effect on downstream neurons, which affects reward responses and behavioral sensitization.^150^ This dual regulatory mechanism is particularly evident during opioid abuse (e.g., morphine and heroin), which can dysregulate mood and reward systems, exacerbating depression-like behaviors. KOR activation is closely associated with aversive emotions and anhedonia in the stress response. Recent clinical studies have suggested that KOR activity can predict the severity of pleasure deficits in schizophrenia.^151^ KOR activation in NAc inhibits excitatory inputs to PV neurons, leading to an imbalance in feedforward inhibition. This mechanism is thought to contribute to the modulation of anhedonia in depression.^152^ In addition, CSDS (Figure 1) inhibits the excitatory output of PV neurons in the region from the claustrum (CLA) to the PrL through a KOR-mediated signaling pathway, leading to PL microcircuit dysfunction through the inhibition of pyramidal neurons.^16^

Opioid receptor-specific modulation of PV neurons offers valuable insights for the development of opioid receptor-targeted therapies for depression. KOR antagonists have been shown to be effective in reducing stress-induced negative emotional responses, thereby alleviating depression-like behaviors.^153^^,^^154^ Additionally, MOR agonists, such as buprenorphine, have been found to improve mood in certain patients.^155^ As a partial agonist, buprenorphine also exhibits KOR antagonistic properties, which contribute to emotional stability while reducing the risk of addiction.^156^ These findings provide new directions and evidence for the use of opioid receptor-targeted therapies in depression treatment.

### Summary of molecular mechanisms

Although the molecular mechanisms discussed above are diverse, they appear to converge on several common features of PV neuron dysfunction in depression. Epigenetic alterations may affect transcriptional programs involved in PV neuron identity and maturation, whereas impaired neurotrophic signaling, particularly involving BDNF/TrkB-related pathways, can compromise PV neuron maintenance, synaptic integration, and plasticity. Ion-channel abnormalities may weaken the FS properties required for PV neurons to synchronize local networks. In parallel, neurotransmitter and receptor imbalances involving glutamatergic, GABAergic, serotonergic, dopaminergic, cholinergic, and opioid systems can alter PV neuron excitability, inhibitory output, and function within emotion-related circuits. Oxidative stress, inflammatory signaling, and PNN disruption may further reduce the structural and metabolic stability of PV neurons. Together, these mechanisms may lead to altered PV expression, impaired PV neuron maturation, weakened inhibitory control, disrupted gamma oscillations, and E/I imbalance in corticolimbic and subcortical circuits. Thus, PV neuron dysfunction in depression may reflect the convergence of multiple stress-sensitive pathways.

## Neural circuit mechanisms

PV neurons are widely distributed across cortical and subcortical brain regions, including the PFC, hippocampus, basolateral amygdala (BLA), NAc, TRN, and interpeduncular nucleus, many of which are implicated in emotion regulation, cognition, and reward-related behaviors. The dysfunction of PV neurons in depression involves not only alterations in their molecular mechanisms but also deficits in their ability to regulate neural networks. PV neurons help maintain the E/I balance by integrating inputs from various brain regions and by sending inhibitory signals to local neurons.

### Medial PFC (mPFC)

Dysfunction of the mPFC, a key upstream brain region involved in emotion regulation that integrates and transduces signals, plays a crucial role in the development of stress-induced depressive-like behaviors.^157^ Under depressive states, PV neurons in the mPFC exhibit complex and dynamic changes in their expression and activity, with no consensus of these alterations. Although PV neurons make up less than 2% of the total neuronal population in the mPFC, they constitute about 40% of the GABAergic interneuron population.^158^ Notably, PV neurons are present in all cortical layers except layer 1 (L1), highlighting their critical role in the regulation of the E/I balance within the cortex, as well as in cognitive and behavioral functions.

Most current studies suggest that depression models, particularly those involving adolescent stress, result in a significant reduction in the number, activity, density, and dendritic complexity of PV neurons in the mPFC, especially in the IL region.^159^ Ketamine has been shown to prevent stress-induced dendritic spine elimination, partly by increasing PV-neuron activity.^160^ The mediodorsal thalamus (MD) projects to PV neurons in the mPFC. Inhibition of MD activity leads to a marked decrease in GABAergic signaling in the mPFC and an increased E/I imbalance, resulting in cognitive deficits and anxiety-like behaviors.^161^ Furthermore, CSDS preferentially impairs the excitatory output from the CLA to PV neurons in the PL, leading to microloop dysfunction in the PL by disrupting the inhibition onto pyramidal neurons (Figure 1).^16^ Thus, PV neurons primarily modulate local circuit activity in the mPFC through their inhibitory synapses onto pyramidal neurons. These pyramidal neurons, in turn, project signals to the amygdala and other areas of the limbic system, influencing mood and cognitive function.

**Figure 5:**
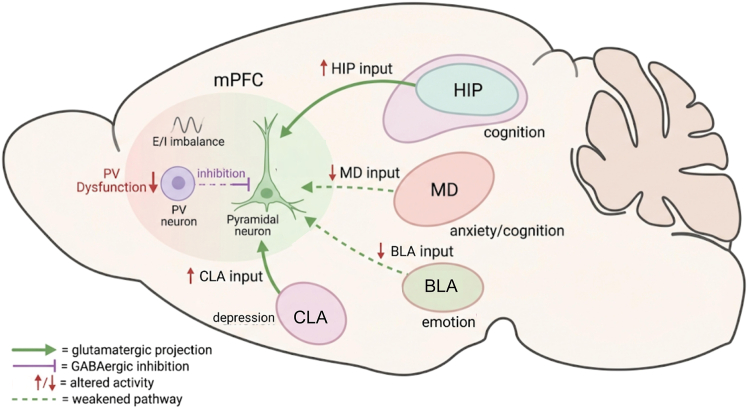
Diagram illustrating the neural projections and associated functions of parvalbumin (PV) neurons in the medial prefrontal cortex (mPFC) PV neurons within the mPFC finely modulate the excitatory-inhibitory balance in the mPFC by projecting to glutamatergic (Glu) neurons and forming local microloops. The mPFC serves as a critical hub integrating excitatory (glutamatergic, green) inputs across key brain regions involved in emotion regulation and cognition. Including the claustrum (CLA), associated with depression; the basolateral amygdala (BLA), linked to anxiety and depression; the mediodorsal thalamus (MD), involved in anxiety and cognition; and the hippocampus (HIP), responsible for cognitive processes. This interconnected network underscores the role of PV and Glu neurons in maintaining the balance of excitatory and inhibitory signals in the mPFC, which is critical for regulating mood, stress responses, and cognitive functions in normal and pathological states such as depression.

In contrast to the findings discussed earlier, some studies have suggested that increased activity of PV neurons in the mPFC during depressive states mediates excessive inhibition of the mPFC, contributing to stress-related affective disorders. These changes involve abnormalities at multiple levels, including molecular, cellular, and network regulatory disturbances. First, chronic stress impacts the balance of glutamate and GABA signaling through stress hormones such as corticosterone, which increases the excitability of PV neurons, leading to E/I imbalance within local networks.^162^ Second, chronic stress-induced synaptic remodeling enhances excitatory inputs to prefrontal PV neurons, further driving high-frequency discharges in these neurons.^163^ Notably, it was found that CUMS-induced changes in PV expression were sex-specific. Stress exposure is linked to increased FosB expression in PV neurons, with female mice showing heightened sensitivity to this effect.^164^ After two weeks of CUMS treatment (Figure 1), female mice exhibited significantly greater impairments in affective and cognitive functions compared to males, accompanied by an increase in PV mRNA expression and the number of PV neurons in the mPFC. Depression resistance in male mice is achieved by reducing NMDA receptor expression. Additionally, increased cFos expression in CAMKIIα neurons in the BLA of female mice may enhance excitatory afferents to prefrontal PV neurons.^109^ It has been shown that excitatory projections from the basolateral nucleus of the BLA form monosynaptic connections with the dendrites and somata of PV neurons in the mPFC. These projections are primarily involved in emotion regulation and stress responses, affecting mPFC function through rapid inhibitory feedback.^158^^,^^165^ Meanwhile, projections from the hippocampus, particularly the ventral hippocampus, are associated with memory and cognitive function. These projections can modulate the E/I balance in the mPFC through the direct activation of PV neurons in the mPFC and may contribute to memory impairments observed in depression.^166^ In addition, inhibition of PV neurons in the IL reduced CUMS-induced Fos expression in PrL, BLA, and ventral lateral aqueduct periaqueductal gray matter (vlPAG), and also attenuated depression-like behaviors associated with chronic stress.^167^ Neural projection relationships modulated by PV neurons in the mPFC play a key role in the pathogenesis of depression (Figure 5). In summary, alterations in the activity of PV neurons in the mPFC during depression arise from a combination of multilevel pathological mechanisms, including disruptions in synaptic signaling pathways, neural circuit abnormalities, and inflammatory responses.

### Hippocampus

The hippocampus is a critical brain region involved in memory and emotion regulation, and while PV neurons represent only a small fraction of the total hippocampal neurons, they play a vital role in modulating the activity of pyramidal neurons and granule cells.^168^ Hippocampal PV interneurons comprise several subtypes, including basket cells, axo-axonic (chandelier-like) cells, and bistratified cells. Among these, PV basket cells (PVBCs) constitute a major subtype and are particularly important for generating rhythmic inhibitory postsynaptic potentials that support gamma oscillations through rapid synchronized firing.^169^^,^^170^ Dysfunction of hippocampal PV neurons has been implicated in mood- and cognition-related abnormalities, potentially by disrupting excitation-inhibition balance and impairing γ oscillatory activity.^171^

Studies of animal models of chronic stress have shown a significant reduction in the number of PV neurons in the hippocampus compared to controls.^172^^,^^173^^,^^174^ Specifically, PV neurons were found to be reduced in all dorsal subregions of the hippocampus, while in the ventral portion, only the CA1 area and DG were significantly affected.^175^ For instance, CUMS and LPS (Figure 1) induced depressive-like behavior in mice and resulted in a significant decrease in the number of PV neurons in the ventral dentate gyrus (vDG). Defects of PV neurons in vDG lead to depressive-like behavior in mice, which is associated with the upregulation of neuroinflammation.^176^ Conversely, prolonged exposure to stressors such as restraint or social stress decreased the number of PV neurons in the dorsal hippocampal subregions and reduced their PV expression.^116^^,^^177^ Not only that, two studies reported different findings, showing no significant changes in PV cell number or expression under CUMS (Figure 1).^178^^,^^179^ These discrepancies suggest that the type of stressor and the duration of exposure may have differential effects on PV neurons. Moreover, PV neurons play a key role in hippocampal gamma oscillations, and their maturation is more protracted than that of principal neurons. Studies have shown that hippocampal pyramidal cell development is largely complete by postnatal day 21 (PND 21), while PVBCs require an additional 4 to 5 weeks to fully mature in terms of their biophysical properties.^180^^,^^181^ As PVBCs mature, the amplitude and frequency of gamma oscillations increase.^182^ This prolonged developmental window may make PV neurons especially vulnerable to early-life stress. Early neonatal stress has been found to impair the development and function of hippocampal PV neurons, resulting in reduced gamma oscillatory activity and contributing to depression and cognitive memory-related deficits.^67^^,^^183^^,^^184^ Together, these findings suggest that early disturbances in PV interneuron development may produce persistent alterations in inhibitory circuit refinement and network oscillations, thereby increasing vulnerability to later stress-related mood disturbances.

**Figure 6:**
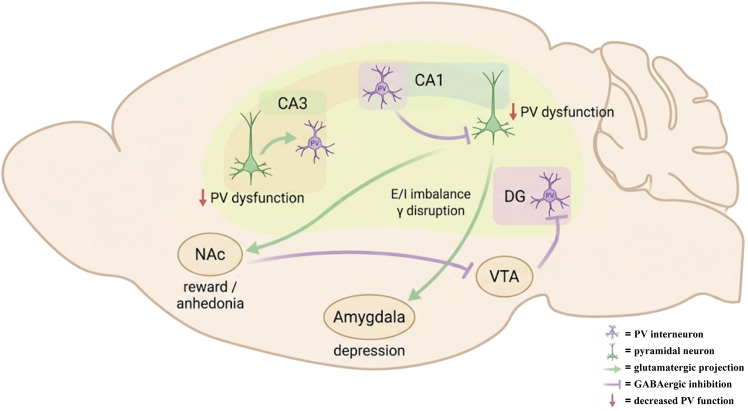
Schematic diagram illustrates the connectivity of hippocampal PV neurons and their projections to mesolimbic circuits In the CA3 region, pyramidal neurons connect to parvalbumin (PV) neurons through glutamatergic projections (purple lines), while PV neurons modulate the activity of pyramidal neurons in the CA1 region via inhibitory GABAergic(γ-aminobutyric acid) projections (orange lines). Furthermore, pyramidal neurons in the CA1 region establish glutamatergic connections with the nucleus accumbens (NAc), a brain region strongly implicated in the pleasure-deficit symptoms characteristic of depression. The NAc influences the ventral tegmental area (VTA) through GABAergic projections. The exact mechanism of neural projections between the VTA and the dentate gyrus (DG) remains unclear (indicated by red dashed lines). This schematic highlights the interaction among the NAc, VTA, and DG, suggesting that these connections may mediate antidepressant effects by modulating PV neuron activity and restoring gamma oscillations.

PV neurons in the hippocampus are involved in modulating neural circuits in depression through neural projections to multiple brain regions (Figure 6). Interference with calcium sensor (CaS) protein expression in the hippocampus impairs short-term synaptic facilitation from CA3 pyramidal neurons to PV neurons, resulting in cognitive impairment in animal models of depression.^185^ Moreover, PV neurons in the ventral hippocampus (vHipp) form synapses with vHipp pyramidal cells projecting to the NAc.^186^ The NAc is a key region in the reward circuit, linked to anhedonia, a hallmark symptom of depression.^187^ A study demonstrated that NAc-DBS alleviated depressive-like behavior and reversed reductions in high gamma oscillations and PV neuron activity in the dorsal dentate gyrus.^188^ Medium spiny neurons (MSNs) in the NAc directly target GABAergic neurons in the ventral tegmental area (VTA) via GABAARs,^189^ while GABAergic neurons in VTA form synaptic connections with the granule cell layer in the DG.^190^ Studies indicate that NAc-DBS enhances dorsal DG PV neuronal activity by disinhibiting VTA-DG GABAergic projections. Furthermore, chemogenetic activation of CA1-NAc projections similarly enhanced dorsal DG PV neuronal activity. Therefore, the NAc-VTA-DG and CA1-NAc projections may be jointly involved in the antidepressant effects of DBS-NAc.^191^ Furthermore, recent studies also suggest that PV neurons preferentially innervate hippocampal pyramidal cells projecting to the amygdala.^192^ These neurons modulate emotional memory and responses via their projections to the amygdala. PV neurons in the hippocampus likely play a crucial role in the onset and progression of depression by modulating these key brain regions. Additionally, treatments such as environmental enrichment and antidepressants (e.g., tianeptine, fluoxetine, clozapine) can reverse hippocampal dysfunction induced by social isolation or chronic stress by enhancing the function of PV neurons, thereby ameliorating depression-like behaviors.^193^^,^^194^

### Basolateral amygdala (BLA)

**Figure 7:**
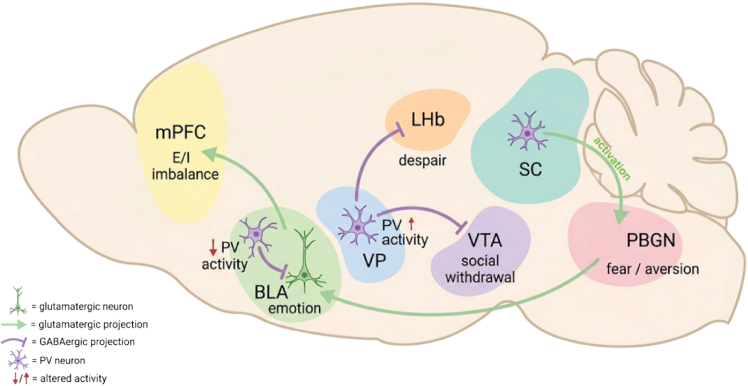
Neural circuitry highlighting PV neurons in the VP and BLA within depression pathways Parvalbumin (PV) neurons in the basolateral amygdala (BLA) interact with local pyramidal neurons via GABAergic projections (purple lines), contributing to the regulation of emotional responses. Pyramidal neurons in the amygdala that receive projections from PV neurons form glutamatergic projections to the medial prefrontal cortex (mPFC) and regulate the excitation-inhibition balance within this region. PV neurons in the ventral pallidum (VP) project to the lateral habenula (LHb) and ventral tegmental area (VTA), influencing distinct depression-like behaviors such as despair and social withdrawal. In the superior colliculus (SC), PV neurons project to the parabrachial nucleus (PBGN), which transmits signals to the amygdala, triggering conditioned aversion and depressive-like behaviors. The red dashed line indicates pathways with undetermined neurotransmitters, highlighting unexplored mechanisms in the connections between brain regions. This figure underscores the critical role of PV neurons in modulating neural circuits involved in mood regulation and stress responses.

The BLA is a crucial brain region for processing emotions, particularly in generating and regulating negative emotions such as fear, anxiety, and sadness. Abnormal BLA function can lead to excessive emotional responses, commonly observed in depression, including low mood, anhedonia, and helplessness.^195^^,^^196^ Approximately 80–85% of neurons in the BLA are pyramidal neurons, and their activity is coordinated through interactions with local interneurons, which make up the remaining 20% of BLA neurons. And PV neurons accounted for almost half of all interneurons in the BLA.^197^ Prolonged stress has been shown to lead to amygdala dysfunction, resulting in decreased activity of PV neurons. Chemogenetic inhibition of amygdala PV neurons induces depressive-like behavior, while chemogenetic activation alleviates the depressive phenotype in mice.^198^ MS medel leads to a decrease in the number of PV neurons in the amygdala of adult rats. However, short-term stress stimuli induce a significant increase in calcium activity in PV neurons in the amygdala.^199^^,^^200^ In addition, the mood of loss can increase the expression of PNNs of PV neurons in the BLA, which increases PV activity and induces loss-like moods similar to depression, including despair and anhedonia.^201^ This difference may stem from the fact that short-term stress triggers adaptive regulation of PV neurons, while long-term stress results in irreversible structural and functional damage. These findings suggest that different types of stress affect PV neuron function in the BLA through distinct mechanisms, thereby influencing emotional states. In addition, PV neurons in the BLA further affect mPFC by inhibiting pyramidal neurons, modulating emotional responses.^202^ In the visual pathway, PV neurons in the superior colliculus (SC) project to the parabrachial nucleus (PBGN), which serves as an intermediary before relaying signals to the amygdala. Activation of this pathway triggers a conditioned aversion response and may also induce depressive-like behaviors.^203^ In summary, PV neurons in the BLA are crucial for regulating emotional responses, and alterations in their activity may represent a key neurobiological basis for depression (Figure 7).

### Ventral pallidum

The ventral pallidum (VP) is central to regulating emotional processing, motivation, and reward. Recent studies show that PV neurons in the VP are key to the CSDS-induced depression phenotype.^204^ During adolescence, social frustration stress leads to an overactive population of PV cells in the VP. Furthermore, optogenetic or chemogenetic inhibition of PV neurons in the VP after stress promoted resistance to social avoidance and despairing behavior in mice, demonstrating a link between PV neurons and depressive-like behavior.^205^ PV neurons in the VP influence downstream regions via different projection pathways. Different PV neurons in the VP project to the lateral habenula (LHb) and VTA, each influencing distinct depression-like behaviors, respectively (Figure 7). PV Neurons in the VP projecting to the LHb show increased intrinsic excitability in depression susceptible animals, and selective chemical genetic inhibition targeting this class of neurons alleviates despairing behavior in depression.^204^ Whereas neurons projecting to the VTA receive more excitatory inputs, and chemogenetic inhibition of these neurons reverses social avoidance induced by CSDS.^204^ Previous studies suggest that VTA DA neuron activity is dysregulated in depression-related states, with both increased and reduced dopaminergic activity reported depending on the stress model and circuit context.^206^ PV neurons in VP projecting to the VTA contribute to CSDS-induced social withdrawal, through the modulation of VTA microcircuits involving both DA and GABAergic neurons.^207^

### Subcortical PV-related circuits

PV-related circuitry in depression extends beyond cortical and limbic interneuron networks to subcortical systems involved in thalamocortical gating and neuromodulatory control. In the TRN, PV neurons play a major role in rhythmic activity and inhibitory gating of thalamocortical communication.^208^ This is relevant to depression because the TRN integrates emotional and attentional signals, including amygdala-related input.^209^ Moreover, peripubertal stress induces depressive-like behavior together with alterations in TRN PV neurons, suggesting that disrupted TRN inhibitory gating may contribute to depressive phenotypes.^210^ However, not all depression-related TRN mechanisms are PV-specific, as some studies implicate SST rather than PV neurons in the TRN-LHb circuit.^211^

Another important subcortical pathway is the medial habenula-interpeduncular nucleus (MHb-IPN) axis. The IPN is largely GABAergic and contains PV neurons.^212^ It receives the major output of the MHb and projects to structures including the raphe nuclei and laterodorsal tegmental nucleus (LDTg).^213^^,^^214^ In addition, the IPN is functionally linked to reward- and aversion-related midbrain circuitry, including VTA-related pathways.^215^ Functionally, the MHb-IPN circuit has been repeatedly implicated in mood regulation, anxiety, and depression-related anhedonia, with circuit hyperactivity associated with anhedonia-like behavior.^216^ Together, these findings support the inclusion of MHb-IPN circuitry in a broader PV-related circuit framework for depression, although direct evidence specifically linking IPN PV neurons to depressive phenotypes remains limited.

## Conclusion

This review examines the molecular and neural circuit mechanisms of PV neurons in depression. First, at the molecular level, PV neuronal dysfunction in depression is closely linked to dysregulated gene expression, epigenetic changes, reduced neurotrophic factors, and neurotransmitter imbalances. Second, PV neurons regulate the excitation-inhibition balance of neural networks by modulating key brain regions, including the mPFC, hippocampus, BLA, and VP. Dysfunction in these regions disrupts emotion regulation and cognitive processes. At the same time, it should be noted that many of the molecular and circuit mechanisms discussed in this review are unlikely to be specific to depression alone. Although PV interneuron dysfunction has emerged as an important theme in MDD, similar abnormalities have also been reported in other psychiatric disorders, particularly schizophrenia. In schizophrenia, PV-related deficits have been most strongly linked to dorsolateral prefrontal cortical microcircuits and are often discussed in connection with gamma oscillatory dysfunction and cognitive impairment. In MDD, by contrast, PV abnormalities are more frequently considered within stress- and emotion-related circuits, such as the medial PFC, hippocampus, amygdala, and reward-associated regions. Therefore, they are more closely tied to affective dysregulation, stress vulnerability, and anhedonia. These observations suggest that PV dysfunction may represent a broader transdiagnostic mechanism affecting shared domains, including E/I imbalance, abnormal network activity, and cognitive dysfunction. Therefore, although the present review focuses on depression, understanding PV dysfunction within a broader psychiatric context may help to better interpret its pathological significance. Although this review focuses on PV interneurons, accumulating evidence suggests that inhibitory dysfunction in MDD is not restricted to a single interneuron subtype. In particular, studies from Sibille and colleagues have consistently implicated SST interneurons in depression, including reduced SST expression across cortical layers and evidence for decreased SST-positive cell density in cortical regions implicated in mood regulation. This is noteworthy because SST interneurons primarily target the distal dendrites of pyramidal neurons and are thought to regulate dendritic integration and associative input processing, whereas PV interneurons mainly provide fast perisomatic inhibition and support temporal precision and gamma-band synchronization. Together, these findings suggest that MDD may involve coordinated disturbances across multiple inhibitory microcircuit components, with PV and SST interneurons contributing in distinct but potentially complementary ways.

Although this review provides insight into the relationship between PV neuron dysfunction and depression, several questions remain to be explored. The measures of PV neuron dysfunction as biomarkers, and future studies should further explore their value in the early identification and intervention of depression. This includes modulating PV neuron activity with drugs or restoring their function through gene editing techniques. Additionally, neuromodulation techniques, such as DBS or TMS, could help restore PV neuron function by modulating the E/I balance in neural circuits, potentially improving depression-like behaviors. In conclusion, PV neurons play an important role in the molecular and circuit mechanisms of depression, and further research may provide a useful basis for the development of more precise therapeutic strategies.

## Acknowledgments

This work was supported by the grants from the 10.13039/501100001809National Natural Science Foundation of China (grant no. 82371237), the 10.13039/501100001809National Natural Science Foundation of China (grant no. 82101298), and the Henan Provincial Key Science Foundation Project (252300421267). Figures are created with BioRender.com.

## Author contributions

Conceptualization, Z.-x.L., T.-e.S., Y.-y.M., W.-d.Z., and J.C.; literature collection and analysis, Z.-x.L., T.-e.S., Q.-r.W., J.-h.Z., W.-z.H., and Y.-y.Z.; writing – original draft, Z.-x.L. and T.-e.S.; writing – review and editing, Z.-x.L., T.-e.S., S.-t.Y, Y.-y.M., W.-d.Z., and J.C.; visualization and figure preparation, Z.-x.L., Q.-r.W., J.-h.Z., and W.-z.H.; supervision, Y.-y.M., W.-d.Z., and J.C.; funding acquisition, W.-d.Z. and J.C. All authors reviewed and approved the final manuscript.

## Declaration of interests

The authors declare no competing interests.
